# Advanced technologies in drowning prevention 

**Published:** 2022-01

**Authors:** Mostafa Golshekan, Ali Davoudi Kiakalayeh

**Affiliations:** ^*a*^ Guilan Road Trauma Research Center, Guilan University of Medical Sciences, Rasht, Iran.

## Abstract

**Background:**

Throughout history, technologies have been created to increase the health of society. In general, technologies have been developed to improve processes and reduce costs. One of the most important issues is the development of new health technologies. Today, the use of nanotechnology, biotechnology, intelligent electronic systems, artificial intelligence, etc. to increase the health of society is very noticeable.

**Methods:**

In the present study, using the data obtained from the Iran National Registry of Drowning (INRD), new methods and technologies in drowning prevention have been studied.

**Results:**

The proposed technologies for drowning are important in three ways: i-To improve processes (increase accuracy and ease of prevention), ii-To reduce the costs (reduction of mortality), and iii- To generate income from new methods (creating a market and producing new equipment). For example, a robotic detector from Coral Detection Systems that scans the pool for drowning activity is a high-tech system for the reduction of mortality.

**Conclusions:**

Finally, it can be said that the development of new equipment and methods in preventing drowning and launching startups in this area is very important to increase not only the level of community health but also the income of the swimming industry.

**Keywords:**

Advanced Technologies, Drowning, Prevention, Iran

